# Supplementary material for: 4-Methylumbelliferone Modulates CAIX to Mitigate Hypoxia-Driven Dysregulation and Enhance PD-1 Immunotherapy in Lung Cancer
Source: Int J Mol Sci. 2025 Oct 27;26(21):10427. doi: 10.3390/ijms262110427 (PMC12610671; doi:10.3390/ijms262110427)
Supplement: Supplementary file 1 [file ijms-26-10427-s001.zip › ijms-3900050-supplementary.pdf]

Supplementary Materials

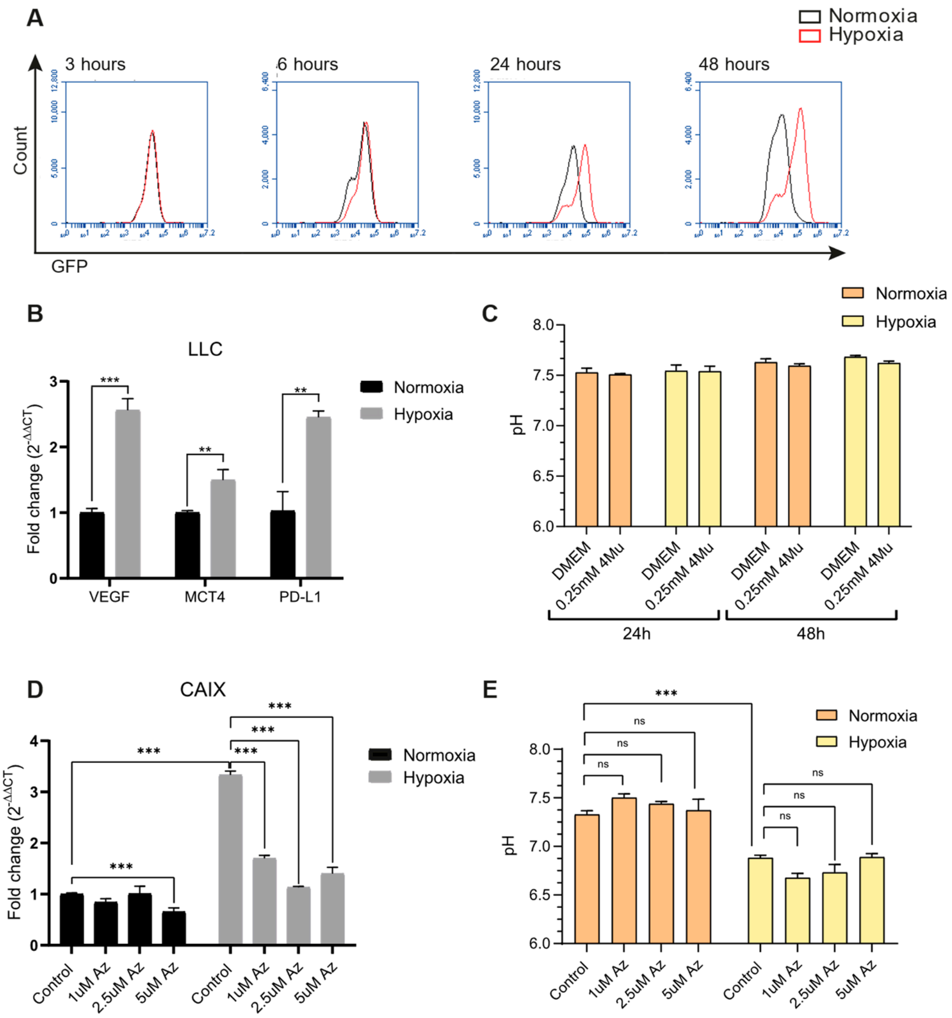

Supplementary Figure S1. Validation of hypoxia-dependent responses. (A) HeLa cells transfected with the HRE/GFP reporter plasmid were used as a reference system to monitor hypoxia responses. GFP fluorescence increased progressively after 24 and 48 h of hypoxic exposure compared with normoxia. (B) In LLC cells, hypoxia upregulated VEGF, MCT4, and PD-L1 mRNA expression, confirming transcriptional activation of hypoxia-regulated genes. (C) Measurement of extracellular pH in culture medium (DMEM) in the presence or absence of 250 μM 4Mu under normoxia and hypoxia at 24 and 48 h. The addition of 4Mu did not intrinsically affect pH, confirming that changes observed in subsequent experiments were due to hypoxia rather than treatment. (D) Relative CAIX mRNA expression was significantly upregulated in hypoxic LLC cells, while hypoxic conditioned media from Az-treated cells showed a significant reduction in CAIX mRNA. (E) Hypoxic exposure also decreased extracellular pH compared with normoxia, but Az is unable to reverse this phenomenon. Data are shown as mean ± SEM of independent experiments; $*p < 0.05$, $***p < 0.001$ versus normoxia.
